# A simple and soft chemical deaggregation method producing single-digit detonation nanodiamonds†

**DOI:** 10.1039/d1na00556a

**Published:** 2022-03-01

**Authors:** Daiki Terada, Frederick Tze Kit So, Bodo Hattendorf, Tamami Yanagi, Eiji Ōsawa, Norikazu Mizuochi, Masahiro Shirakawa, Ryuji Igarashi, Takuya Fabian Segawa

**Affiliations:** Department of Molecular Engineering, Graduate School of Engineering, Kyoto University Nishikyo-Ku Kyoto 615-8510 Japan; Institute for Quantum Life Science, National Institutes for Quantum and Radiological Science and Technology Anagawa 4-9-1, Inage-Ku Chiba 263-8555 Japan igarashi.ryuji@qst.go.jp; Institute of Chemical Research, Kyoto University Uji Kyoto 610-0011 Japan; Laboratory for Inorganic Chemistry ETH Zurich CH-8093 Zürich Switzerland; NanoCarbon Research Institute, AREC, Shinshu University Ueda Nagano 386-8567 Japan; Laboratory for Solid State Physics ETH Zurich 8093 Zürich Switzerland segawat@ethz.ch; Laboratory of Physical Chemistry, ETH Zurich 8093 Zürich Switzerland

## Abstract

Detonation nanodiamonds (DNDs) are a class of very small and spherical diamond nanocrystals. They are used in polymer reinforcement materials or as drug delivery systems in the field of nanomedicine. Synthesized by detonation, only the final deaggregation step down to the single-digit nanometer size (<10 nm) unfolds their full potential. Existing deaggregation methods mainly rely on mechanical forces, such as high-power sonication or bead milling. These techniques entail drawbacks such as contamination of the sample and the need for a specialized apparatus. In this paper, we report a purely chemical deaggregation method by simply combining oxidation in air followed by a boiling acid treatment, to produce highly stable single-digit DNDs in a suspension. The resulting DNDs are surface functionalized with carboxyl groups, the final boiling acid treatment removes primary metal contaminants such as magnesium, iron or copper and the nanoparticles remain dispersed over a wide pH range. Our method can be easily carried out in a standard chemistry laboratory with commonly available laboratory apparatus. This is a key step for many DND-based applications, ranging from materials science to biological or medical applications.

## Abstract 2

Detonation nanodiamonds (DNDs) are a class of very small and spherical diamond nanocrystals. Only after deaggregation they fully unfold their versatile potential from polymer reinforcement materials to drug delivery systems.

## Graphical abstract

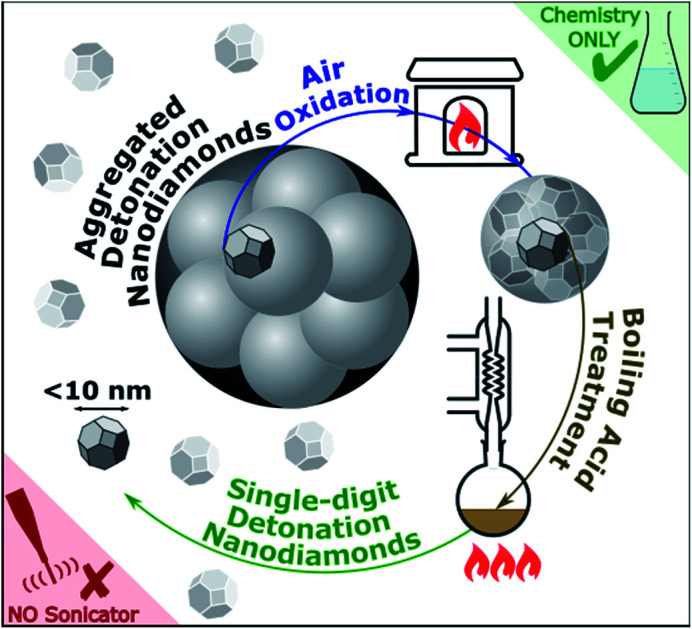

## Introduction

Detonation nanodiamonds (DNDs) are the smallest nanodiamond crystals, having only a single-digit nanometer diameter (<10 nm in size), which can be produced on a large scale *via* an inexpensive detonation method.^1^ In the last two decades, DNDs have received an increasing interest from various fields, such as material or life sciences, due to their extremely small and uniform particle size as well as outstanding physical and chemical properties. As an sp^3^-carbon nanomaterial, DNDs possess remarkable hardness, a high refractive index and the capability of hosting colour defects.^2^ Their application has been widely expanded towards polymer reinforcement materials,^3^ lubricants,^2,4,5^ polishing materials,^6^ antioxidants^7,8^ or sunscreen.^8,9^ Among these applications, DNDs as polymer reinforcement materials have been particularly well investigated. By forming nanocomposites of a polymer matrix and DNDs, nanodiamonds can provide a highly tailorable combination of properties such as superior mechanical, electric or optical properties from the diamond structure, combined with rich surface chemistry and high flexibility for the rational design of the DND–matrix interface.^3^ Further, DNDs are also being regarded as promising carriers in drug delivery systems (DDS), as candidates for disease diagnosis and therapy, as well as imaging probes in the biomedical field thanks to their high biocompatibility.^10–13^ Due to their large surface-to-volume ratio, DNDs have a high drug loading capacity, where the molecules can be attached onto the nanoparticle surface through covalent conjugation or physical adsorption. DNDs enable a prolonged drug retention time by a factor of up to 10 compared to unmodified drugs, resulting in a great enhancement of the chemotherapeutic efficacy.^14^ As a result, DNDs have been promising candidates for *in vivo* applications, applied to a wide range of species for biomedical research, including mice,^15^ monkeys^16^ and humans.^17^ In 2017, DNDs were successfully embedded in a thermoplastic biomaterial for the root canal therapy in humans, revealing a clinically applicable platform of composite biomaterials in the field of nanomedicine.^17^ Last but not least, despite their very small size, DNDs can be a crystal host for nitrogen-vacancy (NV) centers.^18^ NV centers are unique colour centers in diamond with the possibility for optically detected magnetic resonance (ODMR) for potential applications in bioimaging and sensing,^19^ and can be artificially enriched in DNDs.^20^

To utilize the full potential of DNDs, it is extremely important to deaggregate the large DND clusters down to the single-digit nanometer size elementary particles and then to stabilize them in a suspension. For example, it is the size of the drug cargo which determines their behaviours in biological systems. Passive transportation into the cell nucleus is only possible for single-digit nanoparticles. Highly stable single-digit DNDs would therefore allow passive delivery of genetic materials for genetic therapy purposes.^21^ Single-digit nanoparticles can be useful for targeting and for chemical coupling to biomolecules such as proteins or nucleic acids due to their comparable small sizes. Therefore, producing stable single-digit DNDs in a suspension is highly desirable for practically all DND-based applications.

A tentative explanation for the unusually strong DND aggregation is the highly heterogenous chemical surface containing various functional groups, such as carboxyl, hydroxyl, or lactone groups, which may lead to multiple hydrogen bonds and even covalent bonds between adjacent DND particles.^22^ A more homogeneous presence of carboxyl groups on the DND particle surface however would enhance the colloidal stability. Carboxyl groups on the DND surface are formed by oxidation, including air-oxidation^23^ and acid treatment.^2^ A series of other studies see aggregation as mainly triggered by the presence of ions, especially polyvalent metal ions like copper or iron.^24–26^ These experiments strongly supported the latter model, where individual nanodiamonds are coupled through bridging of metal ions and carboxyl groups on the DND surface.^24–26^ Furthermore, a theoretical study highlighted that the DND (100) crystal surfaces have a strongly positive electrostatic potential, while the (111) facets have often a negative one due to graphitization.^27^ In this picture, the electrostatic attraction between DND particles would then lead to strong aggregation.^27^ To date, the aggregation mechanisms have been still under continuous debate and no consensus is yet reached.

Interestingly enough, first deaggregation of DNDs down to a particle size of 4.6 ± 0.8 nm (single-angle DLS, containing 99.4 wt% of the peak)^28^ was only achieved back in 2005 by Krüger *et al.* using stirred-media milling with micron-sized ceramic beads.^29^ Subsequent approaches included ZrO_2_-assisted wet bead milling,^30,31^ bead-assisted sonic disintegration (BASD),^31–33^ salt- and sugar-assisted ball milling,^34^ salt-assisted ultrasonic deaggregation (SAUD),^22^ hydrogen annealing with sonication,^35,36^ and sonication-assisted hydrolysis of oxidized DNDs^37^ (all summarized in Table 1). Although these techniques are able to break the strongly aggregated DNDs into single-digit (<10 nm in size) nanoparticles, they often have critical disadvantages regarding their use in applications, especially in the field of biology. Among all previously mentioned methods, ZrO_2_-assisted wet milling and BASD are the most common techniques. For BASD, micrometer-sized ZrO_2_ particles are accelerated with shock waves generated by the ultrasonic tip. The resulting impact and shear forces from the particle collisions break the strongly aggregated DNDs and create single-digit DNDs in a pH range between *ca.* pH ≈ 3–6.^32,33^ However, the BASD products are accompanied by the difficult-to-remove ZrO_2_ debris contamination and may lead to formation of C–C double bonds and OH functional groups on the DND surface after prolonged BASD.^33^ Although ZrO_2_ particles debris can be reduced from 17 wt% to 9.7 wt% by phosphoric acid treatment and further washing/centrifugation cycles, it is difficult to completely remove the contaminant due to its high chemical resistance and similar particle size to the DNDs.^33^ To address these drawbacks, salt- and sugar-assisted ball milling methods^34^ were developed as alternative techniques utilizing water-soluble crystals such as sodium chloride or sucrose, which can be easily removed from the DND suspension.^22,34^ An attrition mill is indispensable for these techniques. The single-digit DNDs are only obtained at pH ≈ 11 due to the surface profile changes that occurred during the milling process.^34^ As an improved method, SAUD was developed in 2016, using only a homogenizer, glass, metal, or plastic containers and crystalline salts such as NaCl or KCl, enabling simpler production of single-digit DNDs.^22^ By combining the benefits of both, BASD and salt- and sugar-assisted ball milling, SAUD simplifies the disintegration process and overcomes the contamination originating from the beads and does not require a specialized milling chamber. SAUD has, in many perspectives, great advantages over other deaggregation techniques. However, some problems are yet to be solved. Primary metal contaminants are left in the suspension, which may hinder various applications based on these dispersed DNDs. Especially, metal ions adsorbed on the nanodiamond surface have been proved to be a source of cytotoxicity.^38^ An alternative approach was the hydrogenation of DNDs at 500 °C, followed by high-power ultrasonication and centrifugation, which led to monodisperse DNDs in water.^36^ Interestingly, the zeta-potential of these hydrogenated DNDs is strongly positive (>60 mV). In the most recent report by Kume *et al.*, horn sonication was combined with surface chemistry (ozone oxidation and hydrolysis) to enable reduction of sonication power and remove the need of additives during deaggregation.^37^ Although the experimental setup is greatly simplified, the need for a sonotrode (350 W) remains. The strong ultrasound power might damage the sonotrode, which may introduce new contamination into the sample.^33^

**Table 1:** Comparison of existing DND deaggregation techniques (extended from ref. 21)

Techniques	Nature of the method	Experimental setup	Additive	Process	Particle diameter (nm)	Redispersibility, particle diameter (nm)	Remarks	Ref.
Bead-assisted ball milling	Mechanical	Dedicated milling chamber	SiO_2_, ZrO_2_ micro-beads	Strong base or acid treatment to dissolve ZrO_2_ debris	4.6 ± 0.8 (ref. 28)	2300 (after drying)	Difficult-to-remove microbead contamination	28–31
Bead-assisted sonic disintegration (BASD)	Mechanical	High power homogenizer (400–450 W)	ZrO_2_ micro-beads	Strong base or acid treatment to dissolve ZrO_2_ debris	4.8 (for arylated DNDs)^33^	1000–3000 (after freeze-drying) 2300 (after drying)	Difficult-to-remove microbead contamination	32
Annealing in H_2_ gas (500 °C)	Chemical/mechanical	H_2_ gas reaction, high power sonicator		Hydrogenation of the sp^2^ matrix between aggregated DNDs	3–4	—	Strongly positive zeta-potential (>60 mV)	35 and 36
Salt-assisted attrition milling	Mechanical	Dedicated milling chamber	NaCl crystals	Acid treatment to remove iron and other metals, and pH adjustment to 11	<10	16–18 (after drying)	Iron contamination comes from steel balls and parts of the mill	34
Ultra-centrifugation	Mechanical	High power sonicator (500 W)		Centrifugation	4	—	Very low yield	25
Salt-assisted ultrasound deaggregation (SAUD)	Mechanical	High power homogenizer (150 W)	NaCl crystals	Washing/centrifugation	5–10	10–20 (after drying)	NaCl contamination	22
Sonication-assisted hydrolysis of oxidized DNDs	Mechanical/chemical	Dedicated ozone equipment		Hydrolysis of the oxidized surface at pH 9.5	5.9	5.4	Alkaline medium	37
Purely chemical treatment	Chemical	Common chemistry instruments (flask, *etc.*)		Washing/centrifugation	6.9 ± 0.2 (MADLS)	7.1 ± 0.3 (MADLS, after freeze-drying)	Low impurities	This work

**Scheme 1:**
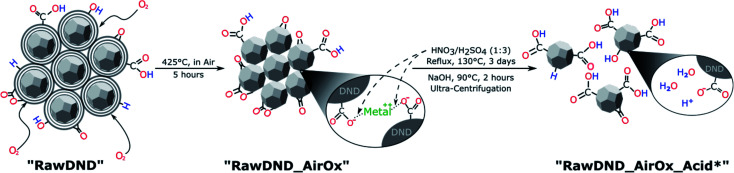
Reaction scheme for the purely chemical deaggregation of detonation nanodiamonds and their abbreviations used throughout the text (for more details, see Scheme S1†).

**Fig. 1:**
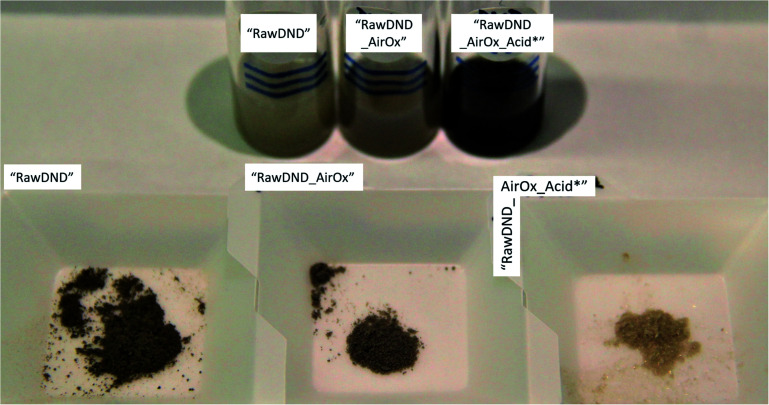
Photograph of suspensions and dried powders, arranging from left to right are, “RawDND”, “RawDND_AirOx” and “RawDND_AirOx_Acid*”. Dark colour of “RawDND” is lighter after air oxidation and finally the sample turned into a shiny light-yellow-grey powder with an extremely feathery texture after boiling acid treatment.

To summarize, conventional mechanical techniques in fabricating a single-digit DND suspension have the following three issues: (1) contamination (beads, metals such as iron, *etc.*), (2) difficulty in controlling the DND surface profile and (3) need for a dedicated apparatus. In this report, we propose a novel, facile, inexpensive technique to create a monodisperse single-digit DND suspension out of pristine DNDs in the form of large aggregate clusters that are commercially available with strongly reduced contamination, using only chemical means: air-oxidation at 425 °C for 5 hours followed by a boiling acid treatment in HNO_3_/H_2_SO_4_ (1 : 3 v/v ratio) at 130 °C for 3 days (Scheme 1 and Fig. 1). As a work up, larger aggregates are separated by ultra-centrifugation. This two-step reaction is inspired by our previous work,^20^ where electron irradiation of nanodiamonds followed by the same boiling acid treatment leads to monodisperse single-digit nanodiamonds. Since the electron irradiation in air had oxidized the nanodiamond surface,^20^ we have replaced this step with a common oxidation step in air. Our new approach produces not only DNDs functionalized with the bioactive carboxyl groups, guaranteeing a high reactivity and dispersibility,^39^ it also successfully removes the initial metal contaminants such as magnesium, iron or copper from the surface of DNDs, generated during the detonation production synthesis. Our technique can be carried out in any common chemistry or nanomaterial laboratory and significantly lowers difficulty and cost with respect to all previously developed mechanical methods. Furthermore, this method serves as a proof of principle that DNDs can be deaggregated solely by surface chemistry and sheds light onto the underlying aggregation mechanisms of DNDs. By successfully deaggregating pristine large DND clusters (up to micrometer size) from two commercial suppliers (Adamas Nanotechnologies and Daicel Corporation), we highlight that our method does not depend on a special starting material of aggregated DNDs.

## Experimental section

### Results and discussion

#### Characterization of DNDs: DRIFTS, XPS and LA-ICPMS

**Fig. 2:**
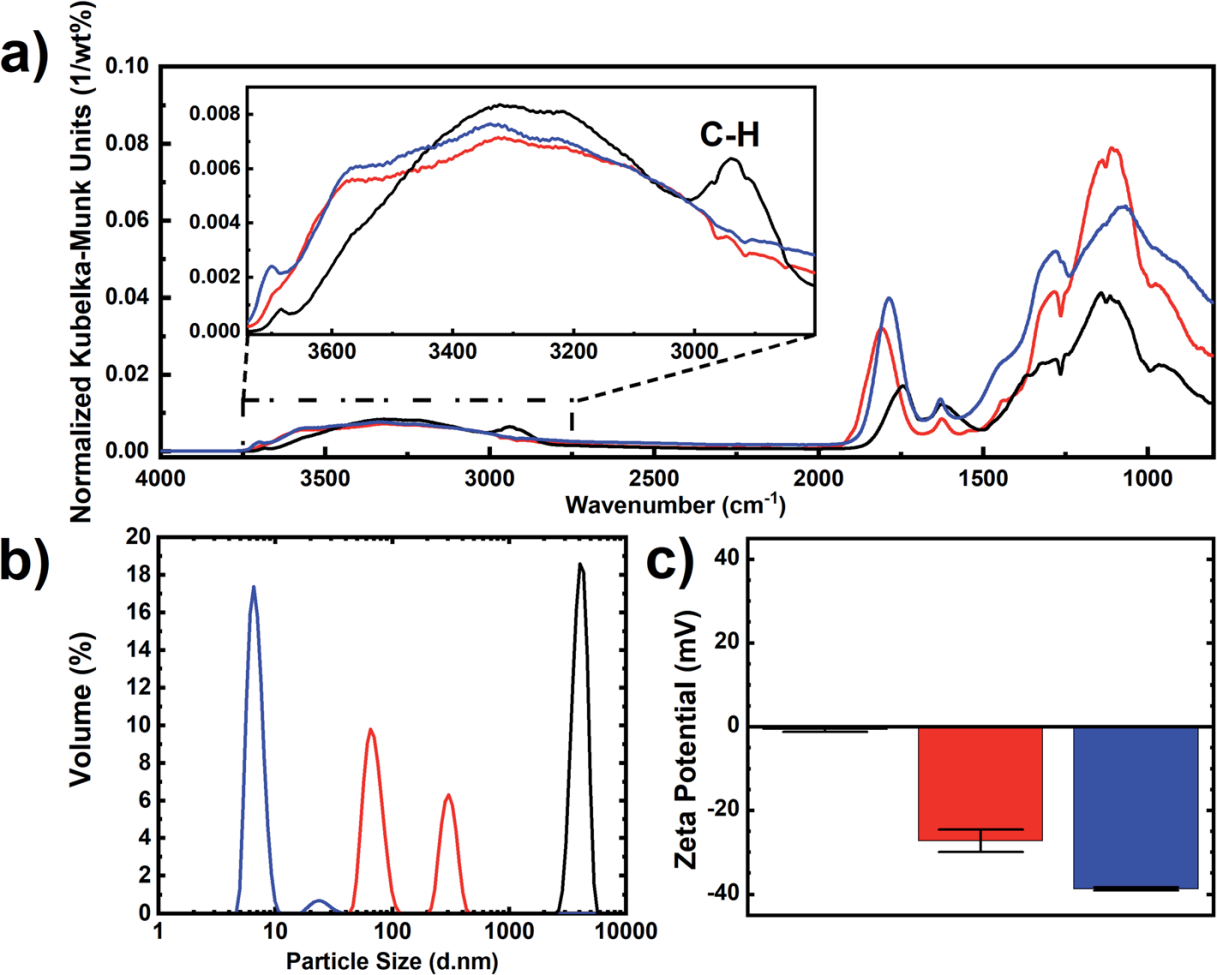
Characterization of “RawDND” (black), “RawDND_AirOx” (red) and “RawDND_AirOx_Acid*” (blue). (a) DRIFT spectra, (b) MADLS size distribution by volume and (c) zeta potentials.

The samples were characterized by diffuse reflectance infrared Fourier transform spectroscopy (DRIFTS) to confirm the organic surface chemical changes during the air-oxidation and boiling acid treatment steps (Fig. 2(a)). As the surface of DNDs adsorbs water molecules from air, the samples were dried at 150 °C for 5 hours to evaporate adsorbed molecules. While C–H vibrations (3000–2850 cm^−1^) disappeared after the air-oxidation, C=O and C–O vibrations (1805 and 940–1370 cm^−1^, respectively) were enhanced, indicating the formation of oxygen-containing derivatives such as carboxylic acids, lactones, anhydrides, hydroxyl groups, cyclic ketones, and saturated structures.^23,40^ After the boiling acid treatment, the C=O vibration peak was downshifted to 1780 cm^−1^, which supports the formation of carboxyl groups on the DND surface.^41^

By consulting a complementary analytical method, we recorded X-ray photoelectron spectra (XPS) of all nanodiamond samples. In the first reaction step of the air-oxidation, the original findings by Osswald *et al.*^23^ were confirmed: An equal sp^2^*vs.* sp^3^ carbon content was fully converted into sp^3^ with no sp^2^ carbon left (see Table 2 and Fig. S2†). In parallel, an increase of C=O, C–O (or C–N) bonds, graphitic carbon and oxygen was observed. While oxidation reactions are expected, the origin of the increase in graphitic carbon is not understood. In contrast, the second reaction step, the boiling acid treatment, did not merely change the ratios of the different carbon groups. Only a slight increase of the sp^3^ carbon going along with a decrease in the graphitic carbon was confirmed. The final product showed contamination with sodium, which is introduced by the NaOH washing step after the boiling acid treatment.

**Table 2:** Relative atomic concentrations (in %) obtained from XPS measurements. The C1s spectra are shown in Fig. S2 (see ESI)

	“Raw DND”	“RawDND_AirOx”	“RawDND_AirOx_Acid*”
Sodium 1s	0.0	0.1	1.6
Oxygen 1s	6.2	13.9	12.6
Nitrogen 1s	1.5	1.7	1.6
C=O	0.6	1.6	1.8
C–O, C–N	2.3	5.8	5.5
sp^3^	44.1	71.9	73.9
sp^2^	44.2	0.0	0.1
Graphite	1.1	4.6	2.9

**Fig. 3:**
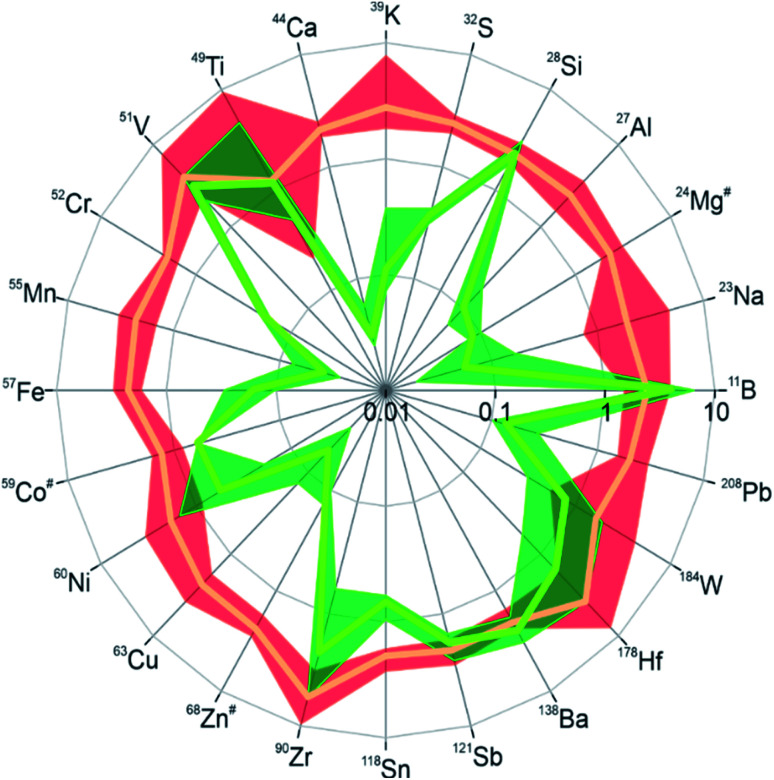
Radar plots showing depletion or enrichment of trace elements in samples “DND_AirOx” (in red) and “DND_AirOx_Acid” (in green) relative to the starting material “RawDND”. Data were obtained by LA-ICPMS analyses of pressed powder pellets. Due to the lack of suitable calibration standards quantification was not attempted. Instead, the ion signal intensities obtained are plotted after normalization to ^13^C intensities for each analysis and in relation to the starting material. The orange and green lines show the mean values from five repeat analyses of each sample. The thicker red and green areas indicate the ranges of the intensity ratios across the five repeats. Values greater than 1 indicate a relative increase in the metal/carbon mass fractions relative to the starting material. Note that the relative intensity ratios are plotted on a log-scale. The ^59^Co ion signals were below the limit of detection (LOD) in samples “RawDND” and “DND_AirOx_Acid” and ^24^Mg and ^68^Zn were below the LOD in the “DND_AirOx_Acid” sample. In these cases (highlighted with #), the ratios were calculated using the respective LOD value.

To cover the inorganic part in the chemical analysis, we performed laser ablation inductively coupled plasma mass spectrometry (LA-ICPMS)^42^ with a special focus on metal impurities in the DND samples. Twenty-four isotopes from different elements were measured and their intensity ratios relative to the carbon-13 signal were determined as a measure of the relative abundance of the respective elements in the material. Due to the lack of a suitable calibration standard only the relative abundance of a specific element across the different samples can be concluded from the data. The results are visualized in Fig. 3, where the intensity ratios of all isotopes are normalized to one for the starting material “RawDND” (numerical values can be found in Table S1†). After the first reaction step of the air-oxidation, almost all elements were observed at intensity ratios two to three times higher than in the starting material. Rather than global contamination, this result is more likely explained by a carbon loss during air oxidation. This quantitatively agrees well with the average weight loss during this experimental step (see Materials and methods). The combustion leads to a partial loss of carbon as CO/CO_2_ while the metal constituents mostly remain in the solid. Their enrichment relative to the matrix element carbon results in a comparable increase in intensity ratios. Notable exceptions are V, Zr and Hf with up to five times increase in intensity ratios, probably by contamination during the combustion process. After boiling the material subsequently in H_2_SO_4_/HNO_3_, substantially lower intensity ratios were obtained for most isotopes indicating that the abundance of these elements could be successfully reduced in the cleaning step. Namely for the elements Na, Al, K, Ca, Na, Cr, Mn, Fe, Co, Cu, and Zn, the average concentration was reduced by more than one order of magnitude. This strongly supports that the second step of the deaggregation is linked to the removal of these metal ions, which hold the individual DND particles together *via* their chelating surface functional groups.^24–26^ The extraction was however not equally efficient and Si, Ti, V, Sb and Ba in particular did not show substantial depletion, while B appeared at even higher levels than after the first reaction step. A similar tendency for the studied elements was previously obtained in an elemental analysis after microwave-assisted purification of detonation nanodiamonds using acid reagents.^43^ This appears to result from a combination of low solubility in the acid mix (Si, Sb, Ba) and potentially the entrainment of B as the contaminant of the reagents used. A comparison with the commercial NanoAmando (NanoCarbon Research Institute) detonation nanodiamonds shows that these BASD (using Zirconia beads) dispersed DNDs have about two orders of magnitude higher Zr contamination than our dispersed “RawDND_AirOx_Acid” (see ESI Table S1†). Earlier studies using inductively coupled plasma-atomic emission spectroscopy (ICP-AES) measured a Zr contamination of about 3 mg g^−1^ in NanoAmando samples.^26^

#### Morphology and crystal structure of DNDs: TEM with SAED, EELS

**Fig. 4:**
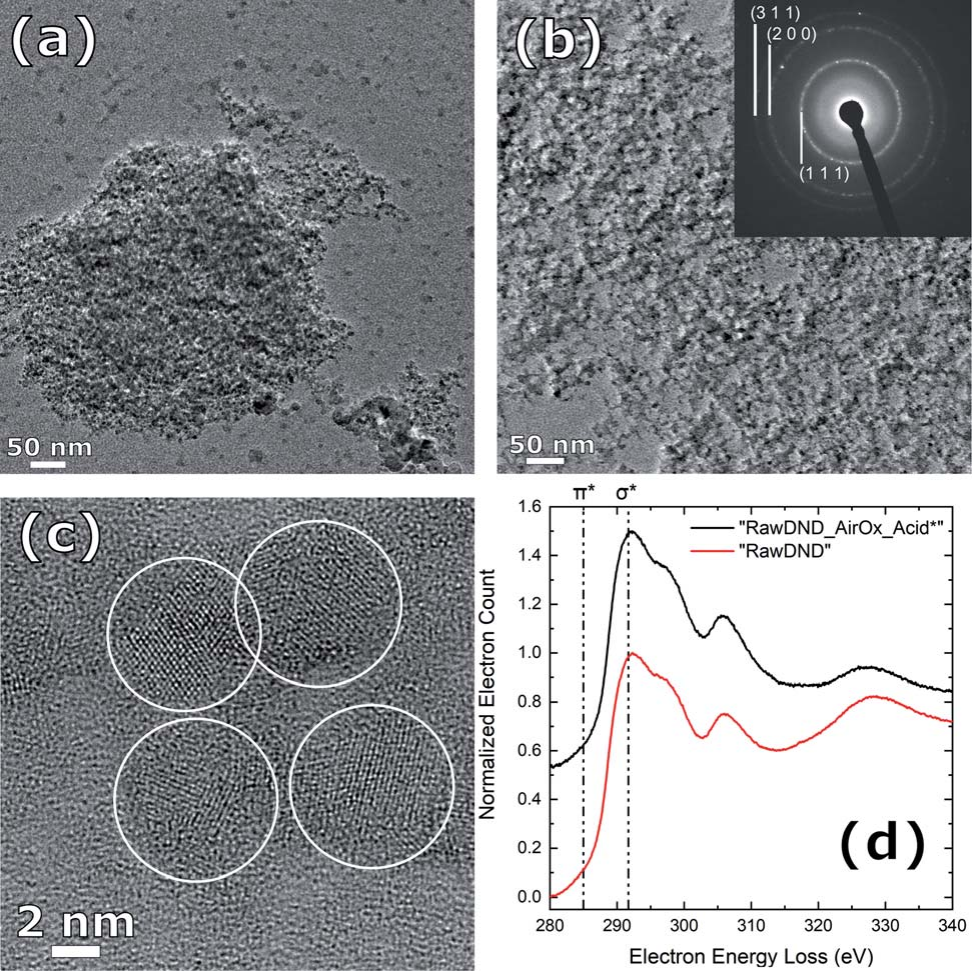
(a) Representative TEM image of the starting material “RawDND” and (b) the final “RawDND_AirOx_Acid*” with the diamond crystal structure confirmed by selected-area electron diffraction (SAED) (inset). (c) Same sample as (b) in a higher magnification, with individual DND particles highlighted with white circles. (d) Normalized electron energy loss spectra (EELS) of the final “RawDND_AirOx_Acid*” (black) and the starting material “RawDND” (red).

To confirm the diamond crystal structure, transmission electron microscopy (TEM) imaging was conducted on the different DND samples (Fig. 4). Fig. 4(a) shows large aggregates in “RawDND” at a low magnification, composed of individual particles of roughly 5 nm (see Fig. S5†). Fig. 4(b) and (c) show the “RawDND_AirOx_Acid*” after air-oxidation and boiling acid treatment in a much more uniform and dispersed state. The selected area electron diffraction (SAED) pattern as an inset of Fig. 4(b), with three rings representing (111), (220), and (311) planes of processed DNDs, confirms that the DND particles retained their nanodiamond crystal structure. Under high magnification in Fig. 4(c), individual nanoparticles in final DNDs with a diameter from 2 to 10 nm were observed, which are highlighted with white circles. In Fig. 4(d), electron energy loss spectra of “RawDND” and “RawDND_AirOx_Acid*” were compared. Spectra from both samples showed a maximum at the 1s to σ* transition (291.7 eV), which is characteristic of diamond sp^3^ carbon. At the 1s to π* transition (285 eV), which appears for sp^2^ and amorphous carbon, no peak was visible in neither of the two spectra.^44,45^ These collective results demonstrated that our method does not influence the crystal structure of the DNDs and that the final product is largely made of sp^3^ diamond carbon.

#### Colloidal stability and redispersibility of DNDs: MADLS and zeta potential

To evaluate the colloidal stability, all samples were investigated by multi-angle dynamic light scattering (MADLS) and zeta potential measurements after dispersion in water. MADLS was used due to the large dispersity of the “RawDNDs” and “RawDNDs_AirOx” samples. As populations might be weakly scattering at a single detection angle, multi-angle measurements provide improved insight into particle size populations. “RawDND” showed a single peak in DLS at 3.5 ± 1.5 μm and a zeta potential around 0 mV (see Fig. 2(b) and (c)) indicating strongly aggregated DNDs. The suspension of the “RawDND_AirOx” shows two peaks centered at 72.1 ± 8.6 nm and 302.3 ± 5.0 nm with a negative zeta potential of −27 mV. The reduction in particle size suggests that the oxygen species on the DND surface enhanced the hydrophilicity and therefore enhanced the dispersibility in water compared to “RawDND”. The simultaneous decrease of the zeta potential supported the presence of oxygen-containing functional groups, which led to an increase in hydrophilicity and hence particle size reduction. After the boiling acid step, the sample reached a monodisperse state with an average hydrodynamic size of 6.9 ± 0.2 nm. The further reduction of the zeta potential down to −40 mV is attributed to the increased number of carboxyl groups. Keeping the results from LA-ICPMS measurements in mind, we believe that the strong reduction of the contamination from many metal elements is at least as important as the organic chemical change on the nanodiamond surface. The results demonstrated that these two chemical steps are needed to make single-digit nanodiamonds. The boiling acid treatment alone does not disperse the starting material “RawDND” (see Fig. S1†). According to these observations, we strongly believe that the stable single-digit state of the sample is contributed by the removal of metal impurities and the presence/formation of the carboxyl groups on the surface of DNDs. The highly negative zeta potential is linked to the presence of carboxyl groups because the COO^−^ groups are formed in water at pH = 5–12.^46^ In contrast, the positive zeta potential is linked to the presence of sp^2^ carbon at the nanodiamond surface.^35^

It is striking that the DLS size distributions of our “RawDND”, “RawDND_AirOx” and “RawDND_AirOx_Acid” correspond remarkably well to the “Secondary aggregates”, “Core agglutinates/Primary aggregates” and “Primary particles” as described earlier by Ōsawa, when using his bead-milling strategy.^28^ Continuing the analogy, our “oxidation in air” would take the place of “intense sonication” (deaggregation down to 100–200 nm), while our “boiling acid treatment” would play the role of the “bead milling” (down to 4.6 ± 0.8 nm by DLS, containing 99.4 wt% of this peak).^28^ Such a comparison of the deaggregation method could further help to understand the mechanistic details of the different approaches.

**Fig. 5:**
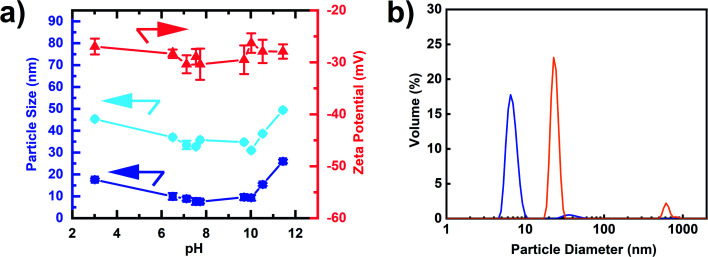
(a) MADLS Volume particle sizes (blue, left axis), DLS Z-average (light-blue, left axis) and zeta potentials (red, right axis) of “RawDND_AirOx_Acid*” at various pH values. (b) Representative MADLS size distribution of redispersed detonation nanodiamonds in water after freeze-drying: “RawDND_AirOx_Acid*” (blue) and BASD-dispersed detonation nanodiamonds NanoAmando (orange).

While all measurements discussed above were performed at pH = 8.75, we further studied the dependence of the colloidal stability as a function of pH (Fig. 5(a)). Aggregation behaviour at varying pH reflects the DND surface profile with functional groups on complex surfaces exhibiting a range of p*K*a values. The single-digit window of the nanoparticle size was 6.5 ≤ pH < 10 with zeta potentials maintained around – 30 mV (Fig. 5(a), numerical values can be found in Table S3†). Strong aggregation was only observed below pH 3, which is likely caused by a loss of surface charge due to protonation of the carboxylate and lactone groups in particular. As a result, the negatively charged DNDs showed good colloidal stability over a wide range of pH values, covering both alkaline and acidic conditions, in comparison to BASD DNDs (positively charged DNDs) as reported before.^47^ Moreover, negatively charged DNDs were shown to have a stronger aggregation resistance at higher ionic strength in comparison to positively charged DNDs.^47^

Finally, we observed that the freeze-dried air-oxidized DNDs after boiling acid were redispersed in water down to a single-digit size of 7.1 ± 0.3 nm (Fig. 5(b)), sharing similar properties to SAUD DNDs, as well as horn sonication oxidized DNDs, but a remarkable difference from freeze-dried BASD DNDs, which cannot be redispersed in the same way and do show aggregation with two peaks of particle sizes 24.6 ± 2.6 nm and 619.4 ± 2.1 nm (Fig. 5(b)). A photograph is shown in Fig. S4.†

## Conclusions

We report a facile, inexpensive chemical approach for the production of monodisperse, single-digit DNDs with reduced metal contamination levels. Our method, oxidation in air followed by a boiling acid (HNO_3_/H_2_SO_4_, 1 : 3 v/v ratio) acid treatment, is “purely chemical”: no high-power sonication or bead milling technique is used for the deaggregation. Thanks to the embedded purification step during chemical deaggregation, this method can be directly applied to the most pristine DNDs. The purely chemical deaggregation method was successfully carried out on two different pristine DNDs in the form of large aggregated particle clusters (up to micrometer size) from two different commercial providers. The total yields were 14 wt% (Adamas Nanotechnologies) and 49 wt% (Daicel Corporation). We assign this large variation to different work-up protocols after the detonation synthesis, which are not disclosed by the manufacturers. Our results are still lower than those by the method with the highest deaggregation yield, which is BASD with a total yield of *ca.* 80 wt%.^33,37^ However, the recipe could serve to provide high purity sp^3^-carbon materials, which could improve the excellent physical/chemical properties of nanocomposites and open up new opportunities in the area of biomedical research as DNDs in DDS, diagnosis and therapy.

The chemical treatments introduce carboxyl groups onto the DND surface, which help stabilize the single-digit nanodiamonds in a suspension by maintaining a highly negative surface charge. Using the platform of a carboxyl enriched nanodiamond surface, the DND could be widely functionalized with readily available chemical nanodiamond modification techniques,^3^ leading to the control of the dispersibility in various solvents and the formation of covalent bonds to polymers of choice.^48^ The produced DNDs are likely to solve two major issues of DND-based bioapplications: (a) cytotoxicity arising from surface metal impurities and (b) reduction of particle size, which is key to cell nucleus targeting and labelling of biomolecules.

Also, carboxyl group enriched DNDs can be immediately conjugated with drugs and biomolecules for in-cell targeting through carbodiimide crosslinking reactions, which would bring a great advantage for using them in bio-applications.^49^ In addition, the single-digit nanodiamonds show excellent colloidal stability over a wide pH range and large ionic strengths. This gives more freedom for the choice of chemical reactions by protecting the nanodiamonds from aggregation.

LA-ICPMS measurements showed that the boiling acid treatment strongly reduces various metal contaminants generated in the detonation synthesis process (see Fig. 3). We believe that this “inorganic clean-up” is the key step to reach the single-digit DND core size by breaking up the inter-particle linkers consisting of metal chelates. Unlike other mechanical methods, DNDs dispersed by our purely chemical method can be simply redispersed in water even after freeze-drying. This is an advantage over DNDs dispersed by BASD, where a subsequent boiling acid step for the removal of metal contaminants (especially Zirconia) leads to aggregation of DNDs.

In summary, the purely chemical technique to successfully produce single-digit nanodiamonds presents solutions for three central problems in the field: (1) strong reduction of metal contamination originating from the detonation synthesis (while avoiding new contaminants through bead milling, *etc.*); (2) creation of a highly negatively charged DND surface with enhanced carboxylic acid groups; (3) no need for specialized setups such as a bead mill or high-power homogenizer. We strongly believe that the use of our single-digit DNDs should provide a standard method for academia and industry, not only for nanocomposite materials, or bioapplications, but for a variety of applications, that are still to be discovered.

## Materials and methods

### Materials

170 nm DND +15 mV positive zeta 1.7% ash (ND Standard 100 g) was purchased from Adamas Nanotechnologies, United States. NanoAmando® Aqueous Colloid Solution (NanoCarbon Research Institute, Ltd., Ueda, Japan) with particle size 3.2 ± 0.6 nm was used as the BASD treated control sample. Nitric acid (60%) and sulphuric acid (95.0+%) were purchased from Fujifilm Wako Pure Chemical Corporation, Japan. Dinnovare Cluster NDs were purchased from Daicel Corporation, Tokyo, Japan. The aggregated detonation nanodiamonds have a size of 200 nm to 10 μm.

### Characterization

DRIFT spectra were acquired by using an FT/IR-6600 (JASCO) instrument equipped with a diffuse reflectance accessory (DR PRO410-M) with a resolution of 4 cm^−1^. The DND samples and KBr (9 : 1, w/w) were ground using a mortar and pestle, and then heated at 150 °C under vacuum for 5 h to remove the adsorbed water. Measurements and the DRIFT spectra processing were conducted, as previously reported.^20^ XPS was measured on a KRATOS ULTRA2 (Shimadzu Corp., Japan) with a Pass Energy of 20 eV and a sample area of 300 × 700 μm, while the charge neutralizer was on. The binding energy was calibrated to N 1s (C–N) = 399.5 eV. For the spectral decomposition of the C 1s signal, an energy difference between sp^2^ and sp^3^ of 1.1 eV was assumed. The C–O, C–N and the C=O signals had an energy of 1.5 and 2.6 eV higher than the sp^3^ signal. LA-ICPMS^42^ was performed at the Laboratory of Inorganic Chemistry at ETH Zurich. The samples were dried for 24 h at 100 mbar and 105 °C, homogenized and finally pressed to pellets (13 mm diameter, 4–6 mm height) under a load of 10 tons for 10 minutes. The pressed pellets were mounted on a microscope slide using double-faced adhesive tape. The mounted samples were placed in the ablation cell of a laser ablation system (GeoLas Q, Coherent, Göttingen^50^) connected to a sector field ICPMS (Element 2, Thermo Scientific, Bremen). The samples were ablated using a fluence of 8.5 J cm^−2^, a pulse repetition rate of 5 Hz and using a crater diameter of 120 μm. All samples were analyzed in time resolved mode using 10 ms integration time per isotope per MS scan. Analyses comprised acquisition of the instrumental background signals for 30 seconds, after which the laser was started and the ablated material was analyzed for 60–90 seconds. The ablation was carried out as single-spot ablation whereby the material was removed from successively deeper regions below the surface of the pressed pellets. Transient signals thus resemble a depth profile, covering approximately 20 μm in depth. Most ion signals from isotopes were recorded using the most sensitive mode of the ICPMS using a mass resolving power (MRP, *m*/Δ*m*) of 300. In order to minimize artifacts from spectral interference however, several isotopes were recorded in a second sequence at a mass resolving power of 4000 (see Table S1†). Data evaluation followed the protocol described by Longerich *et al.*^51^ with the exception that only qualitative data were collected because of the lack of a suitable calibration standard. For LA-ICPMS, the DND sample after boiling acid, but before NaOH washing (“RawDND_AirOx_Acid”) and centrifugation steps was taken. This explains the discrepancy between the Na content obtained from XPS and LA-ICPMS. Hydrodynamic size distributions and zeta potentials of the samples were measured with a Malvern Zetasizer Ultra (Fig. 2(b)) and Zetasizer Nano (Fig. 2(c)) instrument (Malvern Panalytical Ltd.), respectively. In Fig. 2(b), “RawDND”, “RawDND_AirOx”, and “RawDND_AirOx_Acid*” were measured at 1.8, 0.8 and 7 mg mL^−1^ respectively. In Fig. 5, all samples were measured at 7 mg mL^−1^, where the concentration was confirmed by thermogravimetric analysis (TGA-50, Shimadzu Corp). TEM images were taken using a JEM-2200FS + CEOS CETCOR (JEOL) instrument at 200 kV acceleration voltage and 0.1 nm spatial resolution. Samples dispersed in 0.5 mg mL^−1^ Milli-Q water were dried on a germanium (Ge) film with a thickness of 10 nm, and subsequently cleaned with a JIC-410 Ion Cleaner (JEOL) at 300 V.

### Air-oxidation

Raw detonation nanodiamond powder (“RawDND”) from Adamas Nanotechnologies was first ground using a mortar and pestle, and then transferred into a ceramic crucible for surface oxidation in air at 425 °C for 5 hours (AMI-2 Oven, NITTO KAGAKU CO., Ltd), with a yield of 53.8 ± 7.0 wt%. The oven temperature was confirmed using an infrared thermometer (AD-5616, A&D Company, Limited). In the case of aggregated detonation nanodiamond powder (Dinnovare “Cluster NDs”) from Daicel Corporation, identical air oxidization leads to a yield in mass of 94.8 wt%.

### Boiling acid treatment

300 mg of air-oxidized raw nanodiamond powder (RawDND_AirOx) was mixed with 50 mL of nitric acid and sulphuric acid mixture (1 : 3 v/v ratio) in an ice-bath sonicator (Cosmo Bio, Bioruptor UCS-200TM, on/off = 30 s/30 s) for 10 minutes. Next, the mixture was magnetically stirred and heated at 130 °C for 3 days under reflux inside the fume hood. The reaction mixture was then diluted and cleaned with Milli-Q water 3 times by centrifugation at 150 000 RCF for 45 minutes (Beckman Optima Ultra-centrifugation, TLA110 rotor, and TOMY Digital Biology UR-21P handy sonicator) to remove the remaining acid in the solution. The sample was treated with 1 M NaOH solution at 90 °C for 2 hours, and subsequently cleaned with Milli-Q water at the above-mentioned centrifugation parameters until no further de-aggregation was observed (in the case of NaOH treatment, the solution was immediately redispersed into 1 M NaOH after 1 ultra-centrifugation). The black dispersed DND pellet (see Fig. S7†) was gently rinsed, collected, and concentrated along the washes under the same conditions. A white tiny non-dispersed pellet was observed in the pellet. Therefore, in order to remove aggregated particles, the solution was ultra-centrifuged at 10 000 RCF for 1 hour and only the supernatant was collected, with a yield of 29.5 wt% and a final reaction yield of 14 wt%. For the case of aggregated pristine DNDs Dinnovare “Cluster NDs” from Daicel Corporation, the yield after the boiling acid step, taking the supernatant after ultra-centrifugation, was 51.2 wt%, which leads to a total reaction yield of 48.5 wt%.

### Colloidal stability and ionic strength assessment

To examine the colloidal stability under different pH, the final raw DND product (RawDND_AirOx_Acid*) (pH = 7.54) was transferred into two small glass vials separately. 0.5 M HCl solution and 0.5 M NaOH solution were added to the two vials individually using an autopipette, and monitored using a pH meter (HORIBA, laqua 9618s). Once the suspension reached the desirable pH, the hydrodynamic size distribution and zeta potential of the solution were measured using a Malvern Zetasizer Ultra instrument (Malvern Panalytical Ltd.).

## Conflicts of interest

There are no conflicts to declare.

## Supplementary Material

NA-004-D1NA00556A-s001
