# Supplementary material for: A simple and soft chemical deaggregation method producing single-digit detonation nanodiamonds
Source: Nanoscale Adv. 2022 Mar 1;4(10):2268–77. doi: 10.1039/d1na00556a (PMC9418478; doi:10.1039/d1na00556a)
Supplement: NA-004-D1NA00556A-s001 [file NA-004-D1NA00556A-s001.pdf]

This journal is © The Royal Society of Chemistry 2022

Electronic Supplementary Information

Nanoscale Advances (2022)

A simple and soft chemical deaggregation method producing single-digit detonation nanodiamonds

Daiki Terada, Frederick Tze Kit So, Bodo Hattendorf, Tamami Yanagi, Eiji Osawa, Norikazu Mizuochi, Masahiro Shirakawa, Ryuji Igarashi* and Takuya Fabian Segawa*

https://doi.org/10.1039/D1NA00556A

*igarashi.ryuji@qst.go.jp

*segawat@ethz.ch

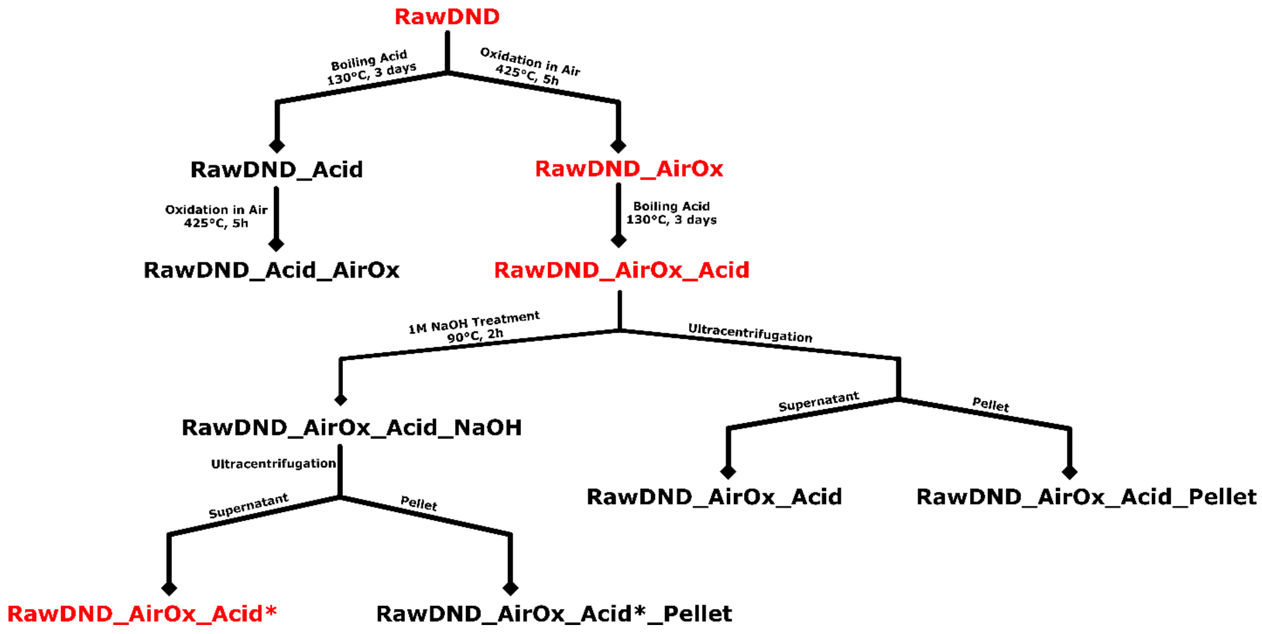

Scheme S1. Overview of all reaction steps and products. The red ones are the starting, intermediate and final products discussed in the main text.

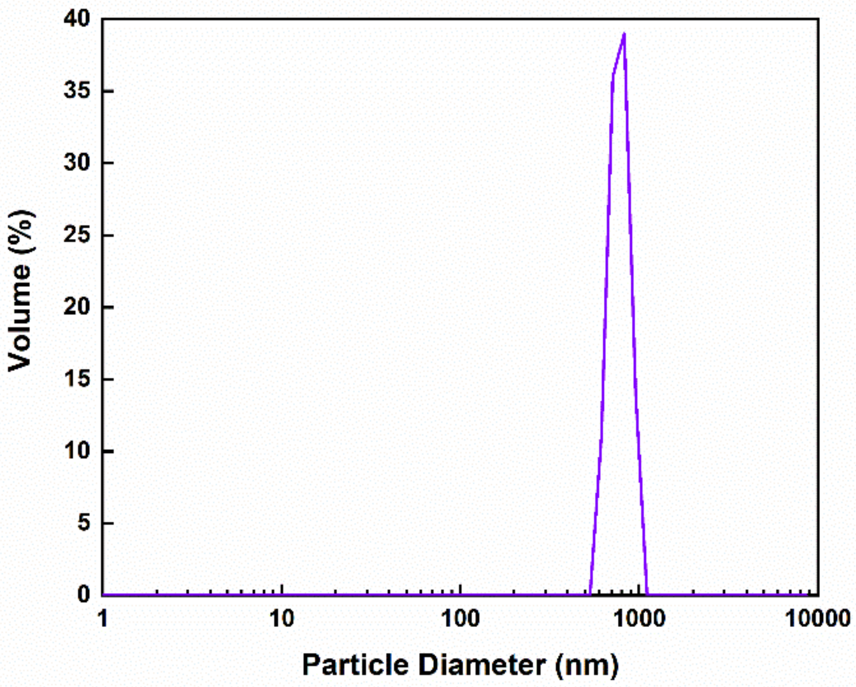

Figure S1. DLS size distribution for “RawDND_Acid” (see Scheme S1) by treatment of the starting “RawDND” with boiling acid only, where a dispersion cannot be reached.

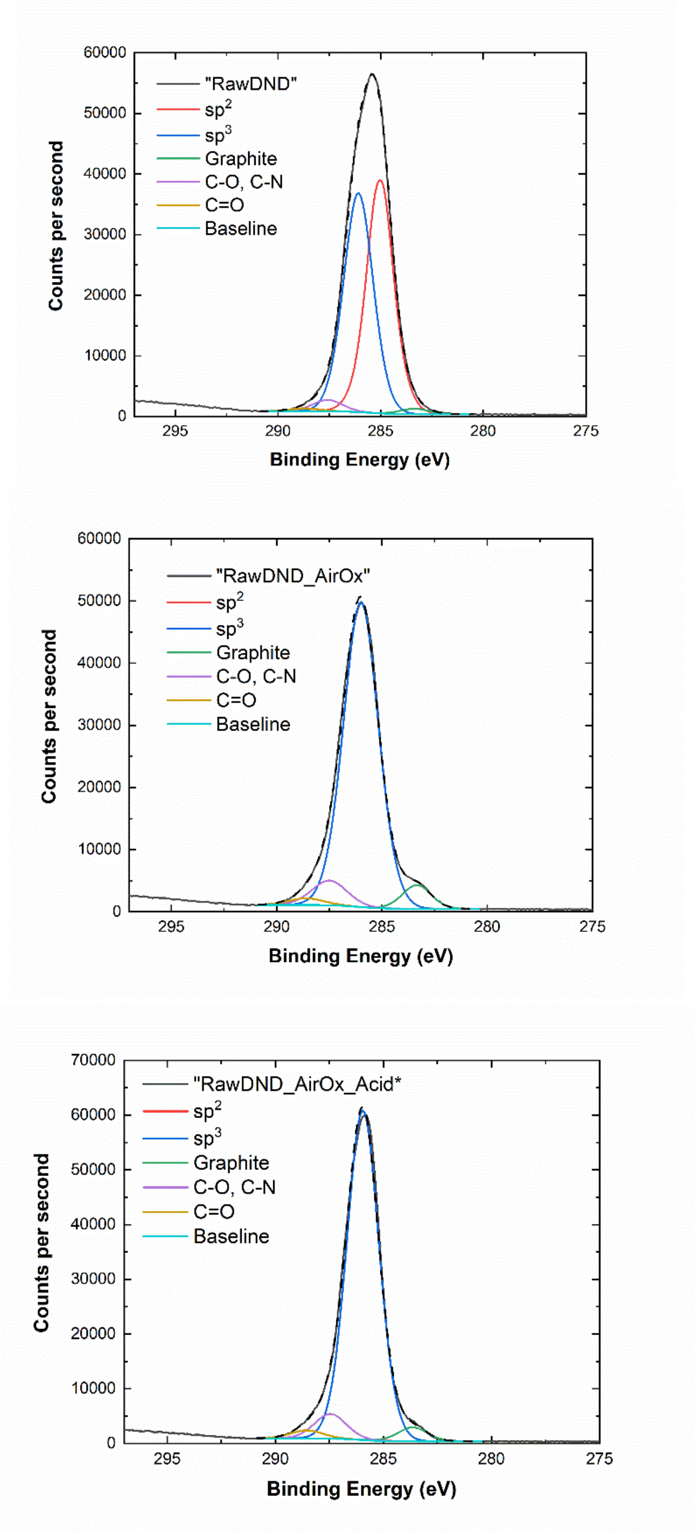

Figure S2. XPS C1s spectra of "RawDND", "RawDND_AirOx" and "RawDND_AirOx_Acid*" with the individual fitting curves.

LA-ICPMS. This method allows for a rapid qualitative comparison of the respective ion signal intensities relative to ^{13}C as reference isotope. One needs to keep in mind however that these intensity ratios do not represent the mass fractions of the element in a sample because the isotopes' sensitivities in an ICPMS vary substantially because of a) isotope abundance, b) ionization efficiency and c) preferred transmission of heavier isotopes. The data can thus only be used to compare the relative abundance of a specific element across the different samples. The resulting intensity ratios are listed in Table S1. Each sample was analyzed at five randomly chosen positions with both MRP settings of the ICPMS. Listed are the mean intensity ratios from the repeat analyses and the resulting standard deviations together with the range observed.

The measurements revealed substantial heterogeneity within each sample not only for the different ablation spots but also within a particular depth profile. An example is given in Figure S3, showing the net ion signals for selected isotope during an ablation. One can observe distinct patterns in the ion signals. While the matrix isotope ^{13}C or trace elements as Ba and Pb do not show substantial variability above the noise throughout the ablation, Ti and Na (less obvious because of the higher base level) show an apparently correlated increase between 40 and 120 seconds of the analysis. This indicates that there is a substantial enrichment ($\approx$ 10 times for Ti in this case) of these elements over a depth of several μm. Zr and Hf on the other hand exhibit a noisy signal structure characterized by numerous, short spikes. Such transient signals are typical for mineral grains of different size, which are present randomly distributed within the bulk. These elements accordingly show a greater spread also in the repeat analyses of the individual samples (Table S1). As a comparison the commercial NanoAmando (NanoCarbon Research Institute) DNDs were measured for comparison. Since their dispersion is performed by BASD using Zirconia beads, the Zr

contamination is about two orders of magnitude higher than in our purely chemically dispersed samples.

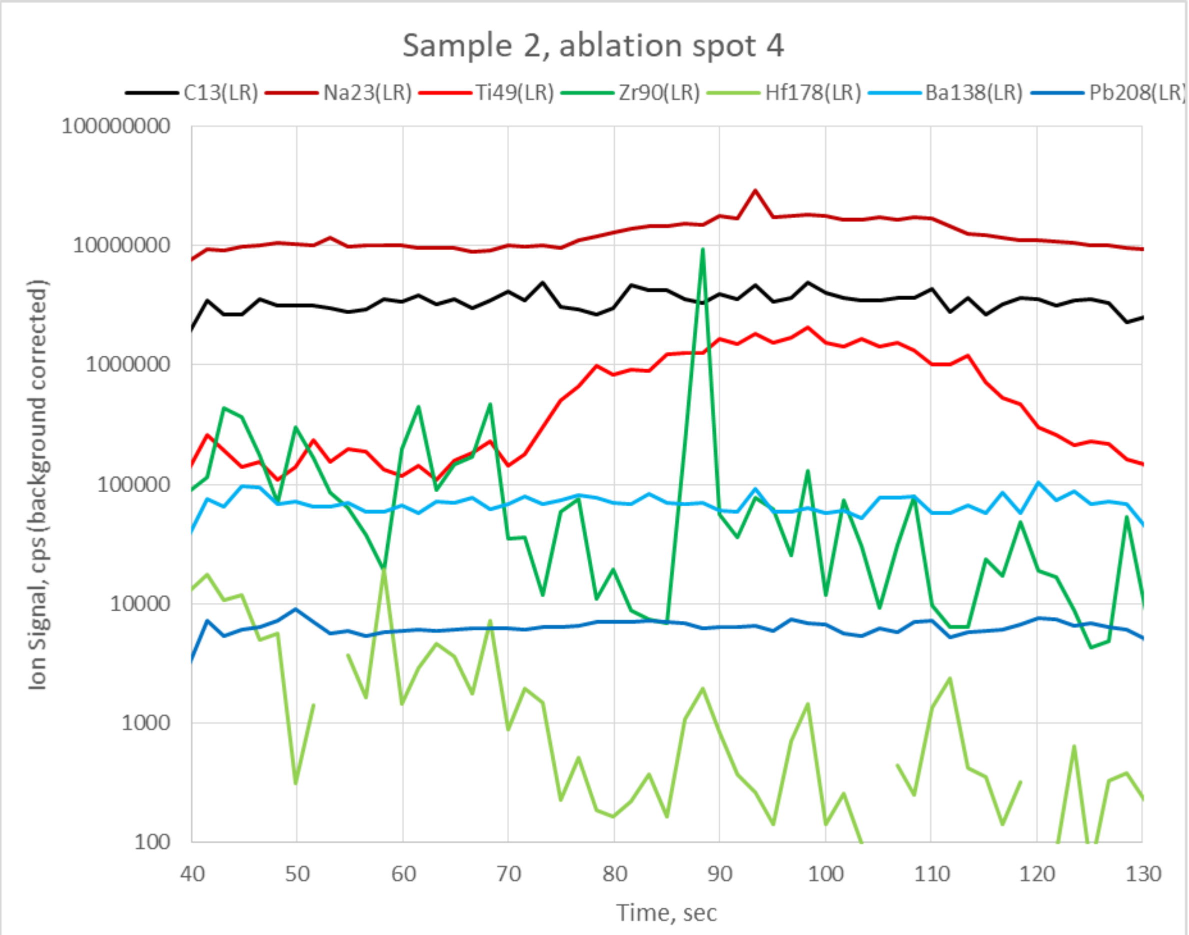

Figure S3. Example for a time resolved LA-ICPMS acquisition of a DND-sample. Smooth signals indicate a homogenous distribution of the elements (e.g. Ba, Pb) across the ablated depth, while gradual changes as visible for example in Ti are stem from large-scale heterogeneities. Short, random spikes in Zr and Hf on the other hand resemble individual (sub-) microns-sized individual particulates present in the material. The inhomogeneous signal could stem from the fact that the measured sample "RawDND_AirOx_Acid" (see Scheme S1) was not yet separated by ultracentrifugation

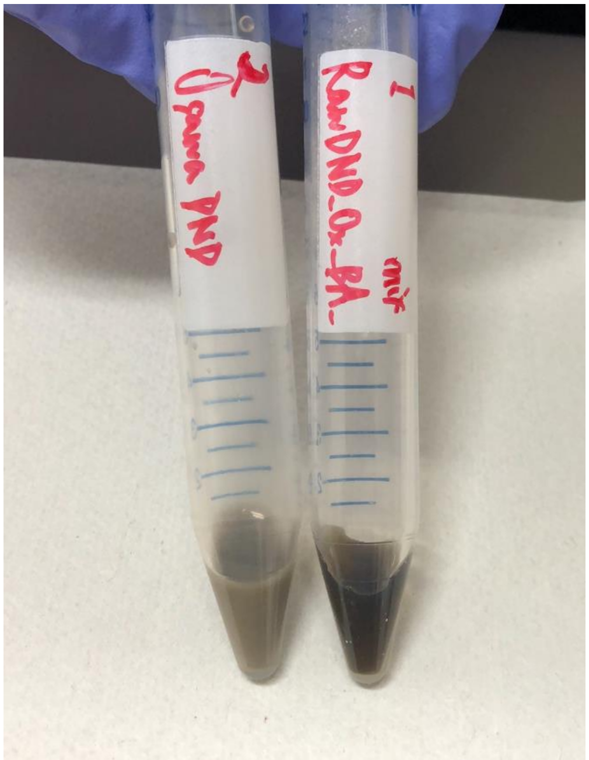

Figure S4. Redispersibility after freeze drying of DNDs

The photograph shows two DND suspensions after freeze drying (corresponding to the size distribution data in Fig. 5b)). On the left: BASD DNDs (NanoAmando®), which cannot be redispersed after freeze drying. On the right: “RawDND_AirOx_Acid*”, which are redispersed down to single-digit size after freeze drying.

Table S1: Intensity ratios obtained by LA-ICPMS relative to ^{13}C as reference isotope

#: net ion signal intensities (10^6 cps), *: Medium resolution measurement (MRP=4000), n.d.: below limit of detection (LOD)

	"RawDND"					"RawDND_AirOx"				
	Mean	SD	max	min	LOD	Mean	SD	max	min	LOD
^{13}C#	3.375	0.040	3.410	3.320		3.16	0.13	3.28	3.01	
^{13}C*#	0.304	0.016	0.321	0.287		0.309	0.023	0.311	0.289	
^{11}B	0.009	0.003	0.014	0.0061	0.00015	0.021	0.001	0.023	0.021	0.00015
^{23}Na	0.8	0.4	1.5	0.5	0.03	1.5	0.5	2.3	1.1	0.015
^{24}Mg*	0.159	0.018	0.174	0.132	0.0018	0.366	0.026	0.404	0.330	0.014
^{27}Al*	0.38	0.08	0.47	0.29	0.044	0.91	0.14	1.06	0.74	0.044
^{28}Si*	1.45	0.16	1.60	1.22	0.008	3.27	0.21	3.53	2.95	0.009
^{32}S*	0.274	0.026	0.309	0.240	0.009	0.664	0.023	0.680	0.622	0.009
^{39}K	0.13	0.05	0.17	0.05	0.018	0.36	0.04	0.40	0.31	0.008
^{44}Ca*	0.067	0.006	0.073	0.058	0.0015	0.143	0.007	0.144	0.136	0.0015
^{49}Ti	0.09	0.09	0.21	0.026	0.00007	0.12	0.08	0.24	0.04	0.0001
^{51}V*	0.0066	0.0020	0.0091	0.0054	0.00025	0.027	0.008	0.040	0.021	0.00027
^{52}Cr*	4.8	0.5	5.7	4.4	0.0015	9.5	0.4	9.7	8.8	0.0024
^{55}Mn	0.22	0.05	0.30	0.16	0.00021	0.51	0.05	0.52	0.45	0.00028
^{57}Fe	0.047	0.007	0.056	0.038	0.0007	0.104	0.007	0.116	0.093	0.0006
^{57}Co	n.d.				0.0008	0.00107	0.00028	0.00130	0.00068	0.0008
^{60}Ni	0.0008	0.0003	0.0014	0.0006	0.00015	0.0015	0.0004	0.0019	0.0012	0.0002
^{63}Cu	0.028	0.006	0.034	0.019	0.0002	0.066	0.006	0.0723	0.060	0.00025
^{68}Zn	0.0043	0.0006	0.0053	0.0040	0.0004	0.0102	0.0006	0.0112	0.0095	0.0006
^{90}Zr	0.013	0.005	0.020	0.009	0.00002	0.072	0.026	0.093	0.0063	0.00004
^{118}Sn	0.0037	0.0005	0.0042	0.0030	0.00007	0.0073	0.0006	0.0079	0.0071	0.00007
^{121}Sb	0.00137	0.00017	0.00155	0.00109	0.00001	0.00287	0.00018	0.00309	0.00264	0.00001
^{138}Ba	0.0112	0.0017	0.0141	0.0100	0.00005	0.0230	0.0011	0.0237	0.0216	0.00006
^{178}Hf	0.00021	0.00006	0.00031	0.00015	0.000006	0.0008	0.0003	0.0012	0.0005	0.00001
^{184}W	0.007	0.005	0.016	0.003	0.000004	0.0107	0.0014	0.0121	0.0084	0.000003
^{208}Pb	0.00102	0.00016	0.00118	0.00075	0.000007	0.00205	0.00015	0.00222	0.00184	0.000008

	"RawDND_AirOx_Acid"					NanoAmando (NanoCarbon Research Institute)				
	Mean	SD	max	min	LOD	Mean	SD	max	min	LOD
^{13}C#	4.14	0.29	4.61	4.03		3.85	0.09	3.99	3.79	
^{13}C*#	0.370	0.017	0.392	0.344		0.313	0.023	0.347	0.295	
^{11}B	0.0352	0.0027	0.0390	0.0337	0.0001	0.0217	0.0007	0.0222	0.0218	0.00015
^{23}Na	0.046	0.026	0.084	0.029	0.01	n.d.				0.01
^{24}Mg*	n.d.				0.01	n.d.				0.01
^{27}Al*	0.038	0.012	0.052	0.030	0.03	1.48	0.13	1.70	1.40	0.03
^{28}Si*	3.2	0.3	3.7	2.9	0.007	0.19	0.06	0.25	0.19	0.008
^{32}S*	0.010	0.005	0.101	0.094	0.007	0.076	0.016	0.10	0.066	0.007
^{39}K	0.014	0.006	0.019	0.012	0.006	n.d.				0.006
^{44}Ca*	0.0021	0.0008	0.0033	0.0018	0.0008	0.0020	0.0012	0.0040	0.0009	0.0009
^{49}Ti	0.109	0.008	0.121	0.105	0.0001	0.0055	0.0011	0.0067	0.0048	0.0001
^{51}V*	0.0200	0.0015	0.0218	0.0186	0.00021	0.0019	0.0005	0.0027	0.0015	0.0002
^{52}Cr*	0.928	0.029	0.960	0.899	0.0024	0.09	0.06	0.14	0.032	0.0018
^{55}Mn	0.0085	0.0021	0.0118	0.0083	0.00017	0.0077	0.0009	0.0089	0.0069	0.0002
^{57}Fe	0.0075	0.0023	0.0113	0.0058	0.0004	0.0227	0.0012	0.0238	0.0224	0.0004
^{57}Co	n.d.				0.0005	n.d.				0.0005
^{60}Ni	0.0005	0.0003	0.0008	0.0003	0.00015	0.0023	0.0005	0.0031	0.0017	0.00015
^{63}Cu	0.0016	0.0007	0.0026	0.0010	0.0002	0.00136	0.00015	0.00161	0.00133	0.0002
^{68}Zn	n.d.				0.0005	n.d.				0.0005
^{90}Zr	0.030	0.024	0.055	0.012	0.000025	6.92	0.29	7.27	6.76	0.00005
^{118}Sn	0.00254	0.00019	0.00281	0.00247	0.00006	0.00143	0.00008	0.00152	0.00134	0.00006
^{121}Sb	0.00251	0.00021	0.00285	0.00234	0.00001	0.000215	0.000014	0.000230	0.000208	0.00001
^{138}Ba	0.0283	0.0028	0.0325	0.0257	0.00004	0.0019	0.0003	0.0024	0.0018	0.00005
^{178}Hf	0.00033	0.00020	0.00056	0.00018	0.00001	0.0820	0.0012	0.0838	0.0809	0.000003
^{184}W	0.0051	0.0003	0.0055	0.0049	0.000002	0.0940	0.0007	0.0947	0.0928	0.000005
^{208}Pb	0.00014	0.00004	0.00020	0.00012	0.000007	0.000520	0.000019	0.000540	0.000509	0.000006

Table S2: Further MADLS result

Sample	Mean (nm) [Average of 5]	Z-avg. (nm)	PDI (DLS)	D50 (nm)	D90 (nm)	D99 (nm)	Concentration (mg/mL)
"RawDND"	3,500 ± 1,500	2,960± 1,830	2.7 ± 3.4	4110.9	4868.5	5486.7	1.8 (Freeze-dry)
"RawDND _AirOx"	69.5 ± 7.6 / 301 ± 4.2	297± 137	0.4 ± 0.2	283.6	358.4	410.7	0.8 (Freeze-dry)
"RawDND _AirOx _Acid*"	6.9 ± 0.2 / 26.5 ± 4.1	35.8± 32.5	0.8 ± 0.6	7.1	22.5	31.0	7 (TGA)
"RawDND _AirOx _Acid*"	5.0 ± 0.5 / 46.3 ± 2.6	44.7 ± 12.8	0.7 ± 0.3	5.0	6.0	58.0	26 (TGA)

Table S3: Further MADLS result on pH measurement (RawDND_AirOx_Acid*), data points in Figure 5a in main context.

(All values were measured at 7 mg/mL, TGA confirmed)

pH	MADLS Peak 1 Volume (nm)		Zeta Potential (nm)		DLS Z-Average (nm)	
	Mean	Standard Deviation	Mean	Standard Deviation	Mean	Standard Deviation
2.4	532.7	41.4	-	-	1245	89.75
2.6	53.8	0.8	-	-	104.9	0.8258
3.0	12.1	0.5	-27.0	1.5	45.3	0.1332
6.5	10.0	1.5	-28.3	0.8	36.99	0.1231
7.1	8.9	0.9	-30.4	1.7	33.51	1.921
7.5	7.6	1.5	-28.9	1.5	32.77	0.1553
7.7	7.5	0.8	-30.4	3.0	35.77	0.1136
9.7	9.6	1.3	-29.5	2.8	34.74	0.1209
10.0	9.3	1.3	-26.3	1.9	30.97	0.1906
10.5	9.4	0.6	-27.9	2.2	38.67	0.2079
11.4	14.1	0.8	-27.9	1.4	49.42	0.2099
11.7	157.3	15.5	-27.3	0.5	188.2	5.196

Table S4: DLS and MADLS measurement conditions

Sample Name	Figure	Concentration (mg/mL)
RawDND	2	0.8 (Freeze-dry)
RawDND_AirOx	2	0.8 (Freeze-dry)
RawDND_AirOx_Acid*	2, 5a	7 (TGA)
RawDND_AirOx_Acid*(Redispersed)	5b	11 (TGA)
BASD Detonation Nanodiamonds (NanoAmando) (Redispersed)	5b	11 (TGA)

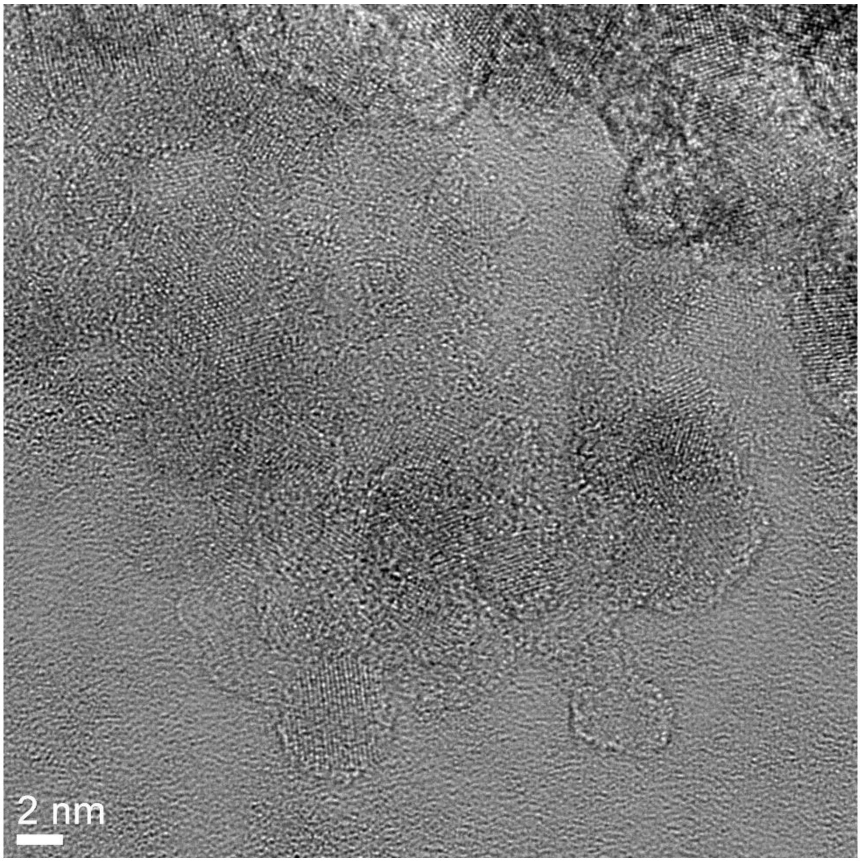

Figure S5. TEM image of initial material "RawDND", illustrating that the large agglomerate particles (around 3.5 µm from MADLS measurement) is composed of individual 5 nm detonation nanodiamonds.

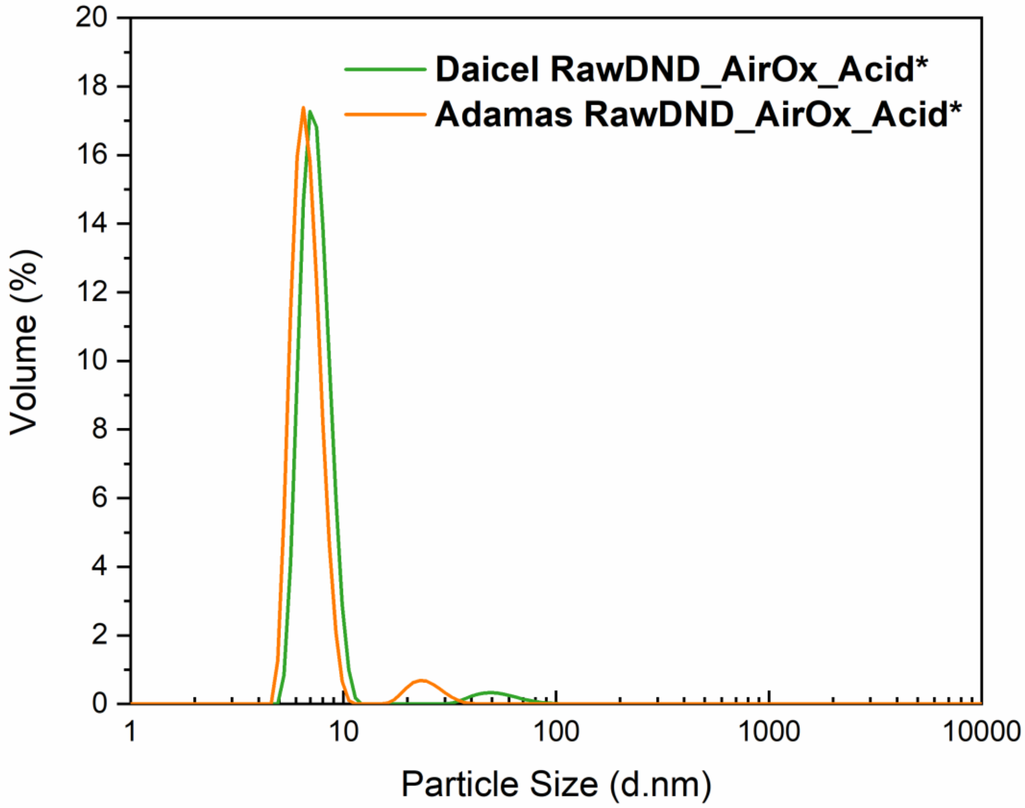

Figure S6. MADLS analysis of "RawDND_AirOx_Acid* from different starting materials, manufactured by Daicel Corporation (Green) and Adamas Nanotechnologies (Orange). The zeta potential of the single-digit DND dispersion from Daicel Corporation was -40.7 +/- 8.0 mV.

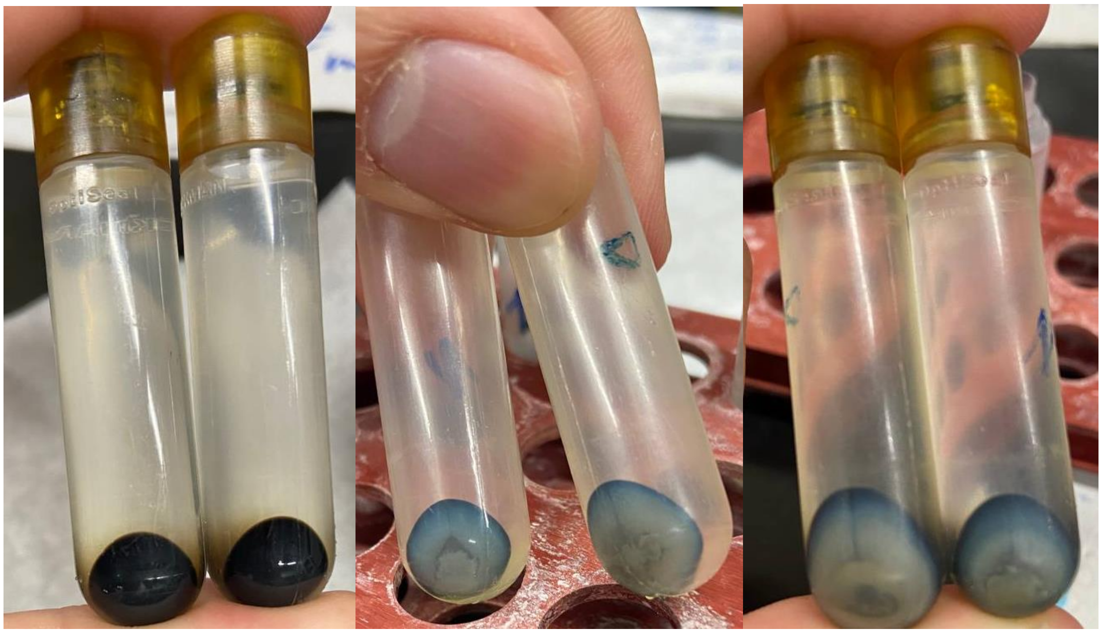

Figure S7. Photos of “RawDND_AirOx_Acid*” deaggregated from Daicel Cooperate “RawDND” (left), “RawDND_AirOx_Acid*_Pellet” synthesized from Daicel Corporation (middle) and from Adamas Nanotechnologies (right).
